# Projected geographic disparities in healthcare worker absenteeism from COVID-19 school closures and the economic feasibility of child care subsidies: a simulation study

**DOI:** 10.1186/s12916-020-01692-w

**Published:** 2020-07-15

**Authors:** Elizabeth T. Chin, Benjamin Q. Huynh, Nathan C. Lo, Trevor Hastie, Sanjay Basu

**Affiliations:** 1 Department of Biomedical Data Science, Stanford University, Stanford, CA USA; 2 Department of Medicine, University of California San Francisco, San Francisco, CA USA; 3 Department of Statistics, Stanford University, Stanford, CA USA; 4 Center for Primary Care, Harvard Medical School, Boston, MA USA; 5 Research and Public Health, Collective Health, San Francisco, CA USA; 6 School of Public Health, Imperial College, London, UK

**Keywords:** School closures, Geographic disparities, Child care, COVID-19, Absenteeism, Simulation study, Geospatial

## Abstract

**Background:**

School closures have been enacted as a measure of mitigation during the ongoing coronavirus disease 2019 (COVID-19) pandemic. It has been shown that school closures could cause absenteeism among healthcare workers with dependent children, but there remains a need for spatially granular analyses of the relationship between school closures and healthcare worker absenteeism to inform local community preparedness.

**Methods:**

We provide national- and county-level simulations of school closures and unmet child care needs across the USA. We develop individual simulations using county-level demographic and occupational data, and model school closure effectiveness with age-structured compartmental models. We perform multivariate quasi-Poisson ecological regressions to find associations between unmet child care needs and COVID-19 vulnerability factors.

**Results:**

At the national level, we estimate the projected rate of unmet child care needs for healthcare worker households to range from 7.4 to 8.7%, and the effectiveness of school closures as a 7.6% and 8.4% reduction in fewer hospital and intensive care unit (ICU) beds, respectively, at peak demand when varying across initial reproduction number estimates by state. At the county level, we find substantial variations of projected unmet child care needs and school closure effects, 9.5% (interquartile range (IQR) 8.2–10.9%) of healthcare worker households and 5.2% (IQR 4.1–6.5%) and 6.8% (IQR 4.8–8.8%) reduction in fewer hospital and ICU beds, respectively, at peak demand. We find significant positive associations between estimated levels of unmet child care needs and diabetes prevalence, county rurality, and race (*p*<0.05). We estimate costs of absenteeism and child care and observe from our models that an estimated 76.3 to 96.8% of counties would find it less expensive to provide child care to all healthcare workers with children than to bear the costs of healthcare worker absenteeism during school closures.

**Conclusions:**

School closures are projected to reduce peak ICU and hospital demand, but could disrupt healthcare systems through absenteeism, especially in counties that are already particularly vulnerable to COVID-19. Child care subsidies could help circumvent the ostensible trade-off between school closures and healthcare worker absenteeism.

## Background

School closures are a common measure of pandemic mitigation for many countries, driven by the logic that social distancing reduces transmission [1–3]. Although school closures are known to reduce transmission, previous works have suggested that school closures could have downstream consequences on the healthcare system such as healthcare worker absenteeism [4, 5].

In the absence of a federal mandate for school closures, the decision of whether or not to close a school is determined by local authorities. However, lack of granular data has restrained previous studies to providing estimates based on state- or national-level data, underscoring the need for more detailed analysis [3]. The needs and capabilities of both schools and healthcare systems vary drastically across the USA, so county-level simulations of healthcare worker absenteeism and school closures could be more impactful and targeted for local communities than state- or national-level simulations. Coronavirus disease 2019 (COVID-19) vulnerability factors such as social determinants of health (e.g., rurality, race) and complicating comorbidities, such as diabetes or cardiovascular disease^1^, vary geographically in the USA, further highlighting the importance of regional analysis [7–9].

To maintain healthcare systems in the event of a school closure, it could be beneficial to assist healthcare workers with child care. Previous work has shown that increased wages are associated with lower absenteeism, so it is possible that child care subsidies could reduce absenteeism by alleviating the financial burden of child care for healthcare workers as well as further incentivizing them to remain at work [10, 11]. Furthermore, the costs of child care (which is the main barrier to finding child care) and the salaries of healthcare workers vary geographically, which would affect both the necessity and the economic feasibility of child care subsidization for healthcare workers in those areas [12].

In previous works, Sadique et al. and Lempel et al. provided national-level cost analyses of school closures under a variety of model assumptions and closure lengths [4, 5]. Bayham and Fenichel provide state-level estimates and include a trade-off analysis on whether closing schools reduces mortality after accounting for disruption to healthcare systems from absenteeism. Given the close trade-off in mortality for school closures and absenteeism, it would be beneficial to explore ways to circumvent the ostensible trade-off through child care subsidies [13].

Here, we provide national- and county-level models that estimate rates of unmet child care needs for healthcare worker households in the event of school closure, the effectiveness of school closures by reduction of peak intensive care unit (ICU) demand, and the ecological association between COVID-19 vulnerability factors and estimated unmet child care needs. We also demonstrate the economic feasibility of child care subsidies as a measure to address healthcare worker absenteeism.

## Methods

### Data

To find county-level demographic and occupational data, we use 5-year estimates from the American Community Survey (ACS) [14] and the Integrated Public Use Microdata Series (IPUMS) [15], a database derived from ACS. The ACS provides comprehensive coverage of data at the county level across factors such as education, housing, employment, and income. For probability estimates of child care dependency, we use data from the National Household Education Survey (NHES) and a Pew Research Center survey on working parents [12, 16]. For county-level estimates of health assessments, we use the Institute for Health Metrics and Evaluation and the CDC Diabetes Interactive Atlas [17, 18]. For county-level fair market rent estimates, we use data from the US Department of Housing and Urban Development [19]. For child care cost estimates, we use data from Child Care Aware of America [20]. We define healthcare workers as individuals belonging to the ACS categories of practitioners (e.g., physicians, nurses, technicians) or support staff (e.g., orderlies, aides, assistants).

### Population simulation

We use individual-level microsimulation models to simulate healthcare workers based on county-level demographic and occupational data for each county in the USA. We obtain estimates of the number of healthcare workers in each county and simulate distributions of them into gender and household type (no children, married with children, single male with children, single female with children) based on existing county-specific household estimates from the ACS. We focus our analyses on households with children ages 6–11 based on age categories provided by ACS.

We seed probabilities of being unable to find child care with data from NHES, Pew Research Center, the US Census Bureau, and IPUMS. Child care arrangements vary significantly based on parental employment, familial relations, between single- and dual-parent households, and gender differences in caretaking of children [21]. In order to simulate which individual in a married couple would be responsible for child care in the event of a school closure, we draw upon survey data from both the Pew Research Center and the US Census Bureau indicating that 89% of working couples rely on the mother for primary child care [16]. We also test sensitivity by using an estimate of 60% instead of 89%.

To simulate ability to find child care in the event of a school closure, we test two different model assumptions:

1. Healthcare workers have difficulty finding child care at the same rates as national estimates. To simulate the probability a worker can find a child care alternative, we draw upon data from the NHES, which found that 50% of households had difficulty finding or could not find satisfactory child care.
2. Difficulty finding child care could be estimated from the household structure of healthcare workers. To simulate household statistics of healthcare workers, we use nationally representative microdata from IPUMS. We take employed healthcare workers who are either the head of the household or the partner of the head of the household and extract the age, relationship, and employment status of each member of the household. We estimate the ability to find child care by identifying other members of a household that could provide care. We define alternative child care as any member within the household that is over 13 and not employed (under 16, unemployed, or not in the labor force). We stratify the data by state, sex, occupation (practitioner or support staff), and partnership status (single or couple) to estimate the state-specific family structures of healthcare workers. We weight these state-specific derived rates of unmet child care need based on county-level demographic information to obtain estimates for each county. As a robustness check, we vary over assumptions as to who in the household can be responsible for child care, including teenagers and grandparents, as well as non-essential workers assumed to be working remotely or newly unemployed (Additional file 2: Table S2, Additional file 3).

Models under the first assumption may provide better estimates in that they include cases beyond household structure (e.g., child care from a relative living elsewhere), but are limited by the assumption that healthcare workers have the same difficulties finding child care as the national average. Models under the second assumption may provide better estimates in that they account for child care difficulties specific to healthcare workers, but are limited by the assumption that all possible caregivers live in the same household as the child.

### Estimating unmet child care needs

We estimate the rate of unmet child care needs for healthcare workers over each county in the USA. Using the probabilities determined in the previous step, we simulate whether or not a given healthcare worker will be able to find alternative child care in the event of a school closure. At both the national and county levels, we draw 1000 simulations from multinomial distributions. We determine unmet child care needs by simulating whether a healthcare worker is the primary caregiver of a household, and whether they are able to find alternative child care in the event of a school closure. We calculate rates of unmet child care needs by dividing over the total number of healthcare workers.

We then repeat the above steps across healthcare worker subgroups (practitioner or support staff) to get a range of estimates. We also perform different estimations based on the different model assumptions proposed in the previous section.

### Transmission models

We model the impact of school closures by county using a compartmental model with an age-structured SEIR framework using the *squire* R package [22]. Transmission events occur through contact between susceptible and infectious individuals. Since rates of contact differ between age groups, we obtain a WAIFW (Who Acquires Infection From Whom) matrix derived for the USA [23]. We assume that school closures will result in a 90% reduction in interactions among children [24]. Since increased household interactions are often cited as an unintended side effect of school closures [25, 26], we also increase interactions between children and other age groups by 10%.

We assume an incubation period of 4.6 days, generation time of 6.75 days, and infectious duration of 2.1 days for mild infections and 4.5 days prior to hospitalization for case infections. [22, 27]. We use state-level estimates of *R*_0_ by state using pre-mitigation estimates of the effective reproduction number (*R*_*e*_) [28]. For each state, we use the earliest *R*_*e*_ and simulate unmitigated growth for 2 weeks. For comparison, we also build models using a static *R*_0_ for all states—the *R*_0_ of severe acute respiratory syndrome coronavirus 2 (SARS-CoV-2) is estimated to be between 2.0 and 6.0, and we examine values within that range (Additional file 2: Table S3). Since the COVID-19 outbreak curve is over a short duration, we ignore births, death, and immigration. Age-stratified hospitalization, critical care (invasive mechanical ventilation, vasopressor support, or further intensive care-level intervention), and infection fatality rates were obtained from Verity et al. [22, 29]. We assume that individuals develop immunity after recovering from COVID-19 in the short term. To simulate transmission and intervention effects at the county level, we seed the simulation with county-level age demographics. Counties were simulated using 100 replicates over 120 days.

### Regression analysis

We perform multiple ecological regression analysis to find associations between unmet child care needs and COVID-19 vulnerability factors. We use a quasi-Poisson regression model with rates of unmet child care needs as the outcome and healthcare worker population as weights [30]. Our factors of interest, based on available county data, are diabetes prevalence, cardiovascular disease mortality, and rurality. We control for race, age, state, household status, sex, population, and fair market rent. We run separate models for cardiovascular disease, diabetes, and rurality, as well as one for controls only.

### Economic analysis

We calculate the economic costs of healthcare worker absenteeism from school closures and compare them to the costs of providing child care to healthcare workers with children. We estimate the percentage of absenteeism from school closures as the percentage of unmet child care needs multiplied by 0.75, since there may be overlap in absenteeism from other factors (e.g., sick leave) or attendance despite unmet child care needs [3]. We estimate the cost of absenteeism as worker wages multiplied by number of workers (split by gender and practitioner/support staff subgroups) within a county, multiplied by 1.4 to account for value not captured by wages, such as taxes, pension, cost of overtime, and paid sick leave [24].

We estimate the cost of providing child care to healthcare workers with children by estimating county-level child care costs and the number of healthcare workers with children per county. We estimate county-level child care costs with a method similar to that used by the Economic Policy Institute’s Family Budget Calculator [31]. Increased costs of providing emergency child care have been estimated to be 1.19–1.23 times the normal rates due to premium pay, hygiene measures, and extra supplies [32]. We multiply the cost of child care by a constant *δ*={1,1.1,1.2,1.3,1.4} to get a range of estimates. We account for healthcare workers who commute across counties (from residence county to workplace county) by using commute flow data from the ACS. We assume the distribution of commuters among the general workforce is equivalent to that of the healthcare workforce, and calculate the income of healthcare workers based on their residence counties, as well as the cost of child care based by workplace counties.

To calculate the cost of a child care subsidy, we multiply the estimated cost of child care by a constant *c*={0.7,0.8,0.9,1}, where *c*=1 denotes a full subsidy and *c*=0.7 denotes a 70% subsidy.

To compare the two costs, we divide the cost of healthcare worker absenteeism from school closure by the cost of providing child care subsidies to all healthcare workers with children at the county level to get a coefficient *ω*. We then calculate the percentage of counties with *ω*>1 at each level of *δ*, indicating the percentage of counties where it is cheaper to provide child care to all healthcare workers with children than it is to bear the costs of healthcare worker absenteeism from school closure. See Additional file 2: Figures S1,2,4 for further details and sensitivity analyses.

## Results

### Estimating unmet child care needs

**Fig. 1:**
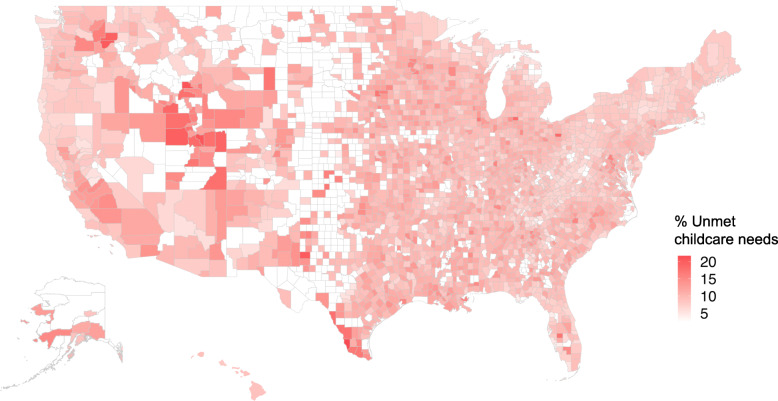
Estimated rates of unmet child care needs for healthcare worker households by county. Counties with confidence interval sizes in the 90th percentile or below are shown

Our national-level simulation based on NHES data provided unmet child care needs estimates of 7.5%, 7.2%, and 7.9% for all healthcare workers, healthcare practitioners/technicians, and healthcare support staff, respectively. Our simulation based on IPUMS data provided estimates of 9.2%, 9.5%, 8.3%. Our county-level approach revealed substantial variation in estimated healthcare worker unmet child care needs across counties, ranging from 2.4 to 23.0% with a median of 9.5% (IQR 8.2–10.9%) (Fig. 1). The model based on IPUMS data had a similar national estimate of difficulty finding child care among healthcare worker households with children (55.3%) as the model based on NHES data (50%) (Table 1).

**Table 1:** Percentage of healthcare worker households with children requiring alternative child care at the national level, across different model assumptions

Household status of HCW	All (%)	NE (%)	GP (%)	OC (%)	OA (%)	Total number
Married/cohabitant mother (all)	57.54	59.88	59.72	78.69	59.06	2,264,875
Single mother (all)	59.34	63.72	62.14	89.16	63.25	680,969
Married/cohabitant father (all)	42.44	44.05	44.35	57.58	43.64	630,269
Single father (all)	63.41	68.20	65.26	84.86	70.79	33,898
Married/cohabitant mother (practitioner)	61.56	63.85	63.66	82.42	62.86	1,596,606
Single mother (practitioner)	59.52	64.15	62.51	89.10	63.49	304,105
Married/cohabitant father (practitioner)	43.03	44.55	44.87	57.73	44.06	540,319
Single father (practitioner)	64.44	68.50	65.63	87.68	70.61	230,40
Married/cohabitant mother (support)	47.93	50.39	50.30	69.76	49.98	668,269
Single mother (support)	59.20	63.38	61.85	89.21	63.06	376,864
Married/cohabitant father (support)	38.89	41.06	41.24	56.73	41.17	89,950
Single father (support)	61.24	67.56	64.47	78.89	71.18	10,858
Total	55.30	57.92	57.55	77.04	57.27	3,610,011

*HCW* = healthcare worker, *NE* = non-essential workers, *GP* = grandparents, *OC* = older children, *OA* = other adults. In healthcare worker households, HCWs can be either mothers or fathers, and either single or married/cohabiting. Support/practitioner refers to the type of healthcare worker. The NE, GP, OC, and OA columns denote non-essential workers, grandparents, older children, and other adults (respectively) in the household who are excluded as potential caregivers

### Transmission models

**Fig. 2:**
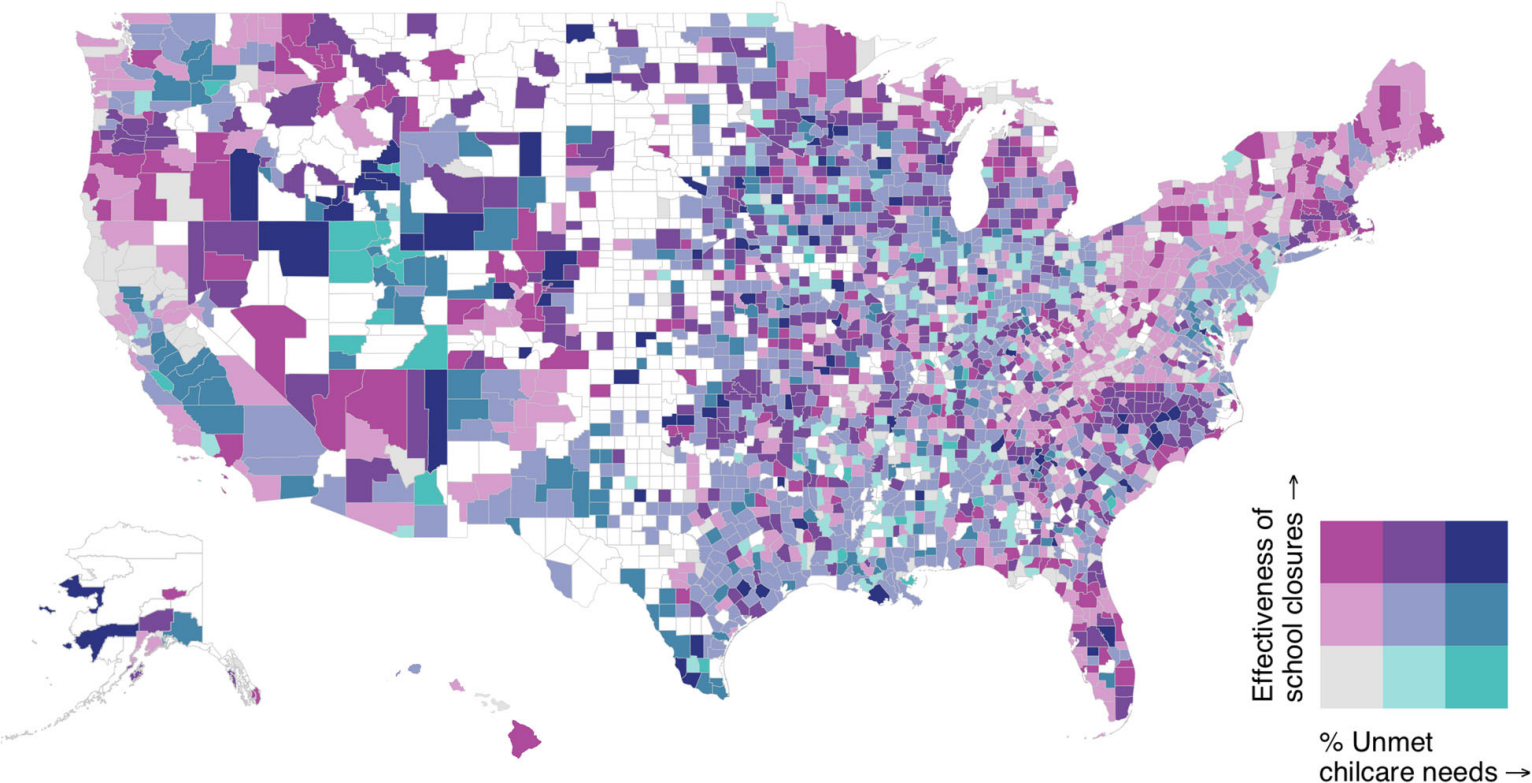
County-level comparison of percent of healthcare worker households with unmet child care needs and effectiveness of school closures using estimated reduction of peak ICU bed demand normalized by state. Counties with confidence interval sizes in the 90th percentile or below are shown. Within-state normalization is used to adjust for different *R*_0_ values across states

Our national-level SEIR model estimated a 7.6% and 8.4% reduction in peak demand for hospital beds and ICU beds, respectively. Our county-level estimates showed substantial variation between counties, with a 5.2% (IQR 4.1–6.5%) reduction in peak hospitalization and 6.8% (IQR 4.8–8.8%) reduction in peak ICU rates (Fig. 2). Our sensitivity analyses show the effectiveness of school closures decreases with increasing *R*_0_ values, which is consistent with past findings [33]. We observe from our models a reduction in hospital demand as a result of school closures with and without increased household interactions (Additional file 2: Table S3).

### Regression analysis

We found from our regression analysis that diabetes prevalence is positively associated with unmet child care needs with a coefficient of 0.22, meaning a 1% increase in diabetes prevalence is associated with a 0.22 percentage point increase in healthcare worker households with unmet child care needs. Cardiovascular disease mortality is negatively associated with unmet child care needs, with a coefficient of less than − 1.859×10^4^. Rurality proportion has a positive coefficient of 0.02, so an increase from non-rurality to full rurality is associated with a 2 percentage point increase in healthcare worker households with unmet child care needs. Proportion of African Americans and proportion of Hispanics have positive coefficients of 0.05 and 0.15, respectively (Additional file 2: Table S4).

### Economic analysis

Based on our values of *δ*, we estimated that for 76.3 to 96.3% of counties, it would be less expensive to provide full child care subsidies to all healthcare workers with children than to bear the costs of healthcare worker absenteeism during school closures (*ω*>1). In the case of partial subsidies (70% of child care costs), 93.3% of counties would find it cheaper to subsidize child care than to bear the costs of absenteeism at *δ*=1.4.

## Discussion

Our models estimated generally high rates of unmet child care needs across different assumptions (> 7%), and our transmission models projected reduced peak hospital and ICU bed demand from school closures. Since it is highly likely that hospital and ICU bed demand would still far exceed capacity for many hospitals [26] despite the effectiveness of school closures, we observe a need for an intervention to reduce absenteeism in the event of school closures. Because we observed large variance of our estimates between counties for all of our county-level analyses, identifying needs and interventions at the county level is likely to be more effective at mitigating harm than a strictly nation- or state-wide strategy.

Our regression analysis estimated that counties with higher percentages of diabetes prevalence, rurality, and Black/Hispanic populations would also have higher rates of unmet child care needs from school closures. Early data have shown that patients with diabetes have higher COVID-19 mortality rates [6] and that African American and Latino communities are disproportionately represented in COVID-19 death counts due to disproportionate representation among essential workers [34, 35]. Furthermore, rural counties are more likely to lack adequate hospital capacity than urban counties [36]. Without a way to mitigate absenteeism, counties that are likely to be most vulnerable to COVID-19 are also estimated to be more vulnerable to absenteeism from school closures, illustrating exacerbated geographic disparities in the absence of adequate child care.

To identify a potential approach to reducing absenteeism, we estimated that a majority of counties (76.3 to 96.3%) could save money by providing child care to their healthcare workers with children in the event of a school closure (*ω*>1). Although it is likely that many child care avenues would also be closed in the event of school closures, subsidized child care costs could still prevent absenteeism by (1) incentivizing work attendance with extra wages and (2) alleviating the financial burden on the entire household, enabling other family or household members to participate in child care.

We observed a number of counties that could be viable targets for child care subsidies based on our estimates (Table 2, Fig. 2, Additional File 1). Counties like Conecuh County, Alabama, and Todd County, South Dakota, have high rates of diabetes, rurality, projected unmet child care needs, as well as a high *ω*, suggesting that they would suffer disproportionately from COVID-19 in the event of school closures, but also that a child care subsidy would be relatively inexpensive for them. Similarly, Hidalgo County, Texas, and Fresno County, California, have high projected rates of unmet child care needs and *ω*, suggesting they are viable targets for child care subsidies. San Francisco County, California, is one of the few counties with *ω*<0.5 (due to high child care costs, low wages, and low projected unmet child care needs), illustrating the variance of our estimates within states. Counties like Bronx County, New York, that have high projected school closure effectiveness but also high projected unmet child care needs could also consider child care subsidies given the large estimated benefit of school closures, but would likely need additional funds since *ω*<1.

**Table 2:** Estimated percentage of healthcare worker households with unmet child care needs, *ω*, closure effectiveness (CE), and actual diabetes prevalence for example counties

State	County	Unmet child care needs (%)	*ω*	CE (%)	Diabetes (%)	Rural (%)
AL	Conecuh	13.2 (13.1, 13.3)	2.24	7.3	20	81
CA	Fresno	12.9 (12.9, 12.9)	3.13	9.4	10	11
CA	San Francisco	3.7 (3.7, 3.7)	0.47	6.8	8	0
NY	Bronx	11.2 (11.2, 11.2)	0.75	9.2	13	0
SD	Todd	19 (18.8, 19.2)	2.93	7.6	15	100
TX	Hidalgo	16.8 (16.7, 16.8)	2.52	9.7	10	5

Closure effectiveness is defined as the percent reduction in peak hospital bed demand when *R*_0_=2. A constant *R*_0_ is used as an adjustment measure to illustrate comparisons of closure effectiveness between counties strictly based on demographics and not levels of social contact

As a simulation study, there are important limitations to our analysis. Simulations rely on assumptions to make predictions, and ours use assumptions derived from available data. For example, we do not know the number of healthcare workers with dependents—we estimate this based on representative data that could be inaccurate for some regions. Similarly, there are no datasets that tell us how many healthcare workers would be unable to find child care in the event of school closures—we instead estimate this using representative microdata. Our assumptions on ability to find child care were derived from pre-pandemic data, which could lead to underestimated absenteeism since child care is harder to find during a pandemic, or an overestimate since newly unemployed or remote workers may now be able to assist with child care. Lack of available data prohibits us from making precise estimates for counties with small populations, or large counties with substantial subcounty-level variation in parameters such as income or child care costs. Given the current uncertainty of transmission parameters, our transmission models should not be used to accurately predict infection and hospitalization rates, but rather to estimate the relative effectiveness of school closures based on the age-demographics of each county. Although our economic analysis demonstrates the affordability of a child care subsidy, our method does not prove that child care subsidies would necessarily reduce absenteeism resulting from school closures. We emphasize that our work does not argue for or against school closures due to currently unclear fatality and transmission data, but rather that we highlight areas that would suffer more in the event of school closure and could therefore benefit more from child care subsidies.

Further research should investigate whether child care subsidies for healthcare workers would reduce absenteeism in the event of school closures from a pandemic, and also how risk of infection may impact healthcare worker absenteeism. Additionally, research efforts should identify how school closures in pandemics impact more vulnerable populations for whom robust data does not currently exist. Further research efforts should also be placed to determine the effect of school closures on the absenteeism of other kinds of essential workers, instead of just healthcare workers.

## Conclusions

Our analyses suggest geographic disparities in unmet child care needs of healthcare workers from school closures, exploring the possibility of targeted child care subsidies for local communities. We demonstrate the economic feasibility of child care subsidies to circumvent the trade-off between school closures and healthcare worker absenteeism in the majority of US counties. Child care subsidies may play a critical role in maintaining the healthcare work force, and such actions could help reduce preventable harm resulting from school closures. Our study provides a step towards informing future work on better understanding the effects of locally targeted non-pharmaceutical interventions in the event of disease outbreak.

## Supplementary information

**Additional file 1** Table 2: County-level data with estimated rates of unmet child care needs derived from IPUMS and NHES data, *ω*, and closure effectiveness, as well as actual rates of diabetes prevalence, cardiovascular disease (CVD) mortality, and rurality.

**Additional file 2** Figure S1: Sensitivity analysis for economic feasibility analysis. Figure S2: Sensitivity of the proportion of counties with *ω*<1 stratified by rurality. Figure S3: County-level comparison of unmet childcare needs and CVD mortality. Figure S4: County-level comparisons of unmet childcare needs and *ω*. Figure S5: County-level comparison of unmet childcare needs and effectiveness of school closures. Table S2: Sensitivity analysis of unmet childcare needs estimates. Table S3: Sensitivity analysis for transmission models. Table S4: Output of ecological regressions. Supplementary information on the economic analysis, figures of bivariate maps, equations of age-stratified compartmental models, sensitivity analyses of unmet child care needs and transmission models, and model output for multivariate association analysis.

**Additional file 3** Table 1: List of ACS codes used to determine essential vs non-essential workers.

## Data Availability

The final processed data analyzed during this study are included in this published article [and its supplementary information files]. The raw datasets are publicly available from the sources described in the text.

## Abbreviations

ACS: American Community Survey
CVD: Cardiovascular disease
COVID-19: Coronavirus disease 2019
ICU: Intensive care unit
IPUMS: Integrated Public Use Microdata Series
IQR: Interquartile range
NHES: National Household Education Survey
SARS-CoV-2: Severe acute respiratory syndrome coronavirus 2
WAIFW: Who Acquires Infection From Whom

## Footnotes

1 China’s Center for Disease Control reported a 0.9% case fatality with no reported comorbidities and 7.3% and 10.5% for comorbidities of diabetes and cardiovascular disease, respectively [6].
