# Supplementary material for: Projected geographic disparities in healthcare worker absenteeism from COVID-19 school closures and the economic feasibility of child care subsidies: a simulation study
Source: BMC Med. 2020 Jul 15;18:218. doi: 10.1186/s12916-020-01692-w (PMC7360472; doi:10.1186/s12916-020-01692-w)
Supplement: Supplementary file 2 — Additional file 2 Figure S1: Sensitivity analysis for economic feasibility analysis. Figure S2: Sensitivity of the proportion of counties with ω<1 stratified by rurality. Figure S3: County-level comparison of unmet childcare needs and CVD mortality. Figure S4: County-level comparisons of unmet childcare needs and ω. Figure S5: County-level comparison of unmet childcare needs and effectiveness of school closures. Table S2: Sensitivity analysis of unmet childcare needs estimates. Table S3: Sensitivity analysis for transmission models. Table S4: Output of ecological regressions. Supplementary information on the economic analysis, figures of bivariate maps, equations of age-stratified compartmental models, sensitivity analyses of unmet child care needs and transmission models, and model output for multivariate association analysis. [file 12916_2020_1692_MOESM2_ESM.pdf]

RESEARCH

Projected geographic disparities in healthcare worker absenteeism from COVID-19 school closures and the economic feasibility of child care subsidies: a simulation study (Supplementary Information)

Elizabeth T Chin*†, Benjamin Q Huynh†, Nathan C Lo, Trevor Hastie and Sanjay Basu

*Correspondence:
etchin@stanford.edu
Full list of author information is available at the end of the article
†Equal contributor

Economic analysis

Here we describe how we obtained county-level estimates of childcare costs and wages. We use state-level child care costs from CCAoA and adjust them to county-level by applying the ratio between state-level and county-level fair market rents from HUD. We calculate state-level rents from HUD by taking population-weighted averages of county rents. To estimate the number of healthcare workers with children at the county-level, we take the state-level proportion of healthcare workers with children from IPUMS and apply it to the county-level number of healthcare workers from ACS. We then calculate the county-level cost of providing child care to healthcare workers by multiplying child care costs by the proportion of healthcare workers with children.

For estimating county-level wages, some counties with low populations had redacted wages to preserve anonymity. We used multiple imputation by chained equations to impute these cases. To get all county-level wages, we multiplied the number of healthcare workers (by occupation group and sex) by their subgroup-respective county-level median wages.

Robustness checks

Here we provide sensitivity analyses and robustness checks of our estimates across various parameters. Table S2 shows different estimates of unmet child care needs based on different assumptions for determining child care needs. Table S3 Shows different estimates of school closure effectiveness based on varying the basic reproduction number from 2 to 6 when holding the reproduction number constant across all states and when varying the basic reproduction number using state-specific estimates across the mean and 95% confidence intervals.

Figure S1 displays the cut off points of rho and delta for 70% of counties to reach $\omega > 1$, where $\rho = \{0.5, 0.6, 0.7, 0.8, 0.9, 1\}$ is the proportion of those with unmet child care needs who go on to be absent from work and $\delta = \{1, 1.1, 1.2, 1.3, 1.4\}$ is the increased cost for emergency child care compared to normal costs. Even under pessimistic parameter assumptions ($\rho = 0.6, \delta = 1.4$), 70% of counties can still afford partial child care subsidies (70%) over bearing absenteeism costs.

Figure S2 shows that the rurality proportions across counties remains relatively constant across values of ρ, suggesting county characteristics do not change across parameter changes.

The list of occupation codes through the American Community Survey that were used to categorize essential workers is included as `Additional file 3: Table S1: Occupation codes for essential worker classification`.

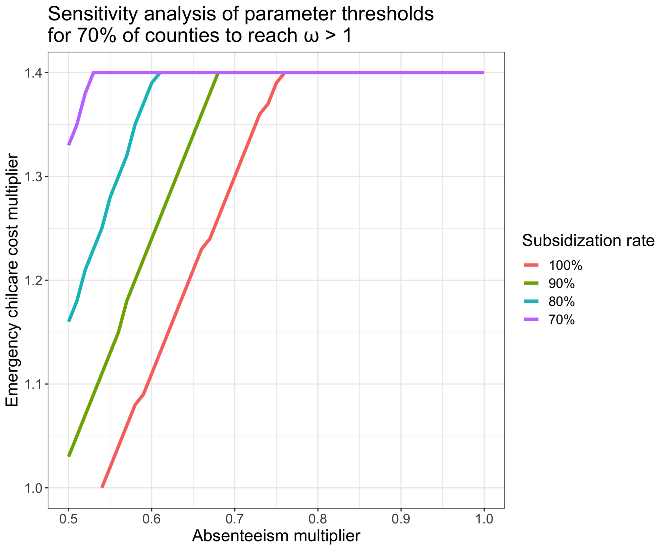

Figure S1: Sensitivity analysis of parameter thresholds for 70% of counties to reach $\omega > 1$. Colored lines indicate different levels of subsidization rates.

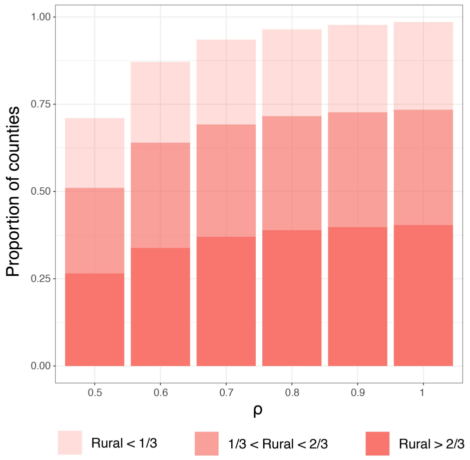

Figure S2: Proportion of counties with higher rates of lost wages due to absenteeism than costs of child care $(\omega > 1)$ across $\rho = \{0.5, 0.6, 0.7, 0.8, 0.9, 1.\}$. Bars are shaded based on the level of rurality of counties.

Additional maps

Absenteeism, complication factors, and wages

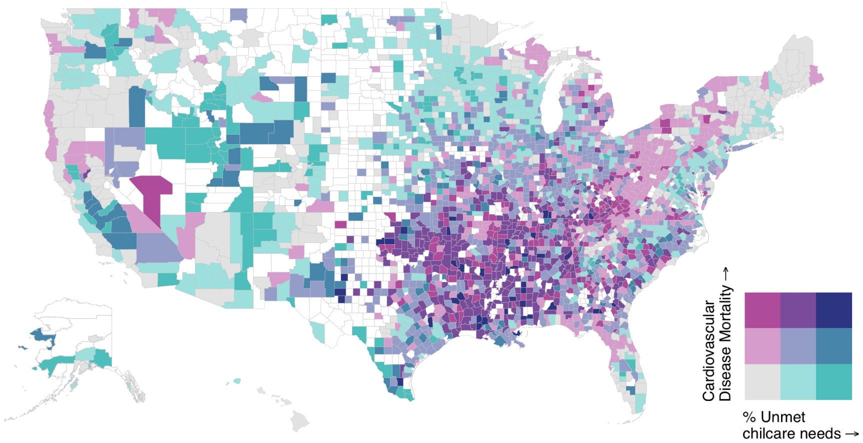

Figure S3: County-level comparison of percent of healthcare worker households with unmet childcare needs and cardiovascular disease mortality (deaths per 100,000 people). Counties with confidence interval sizes in the 90th percentile or below are shown.

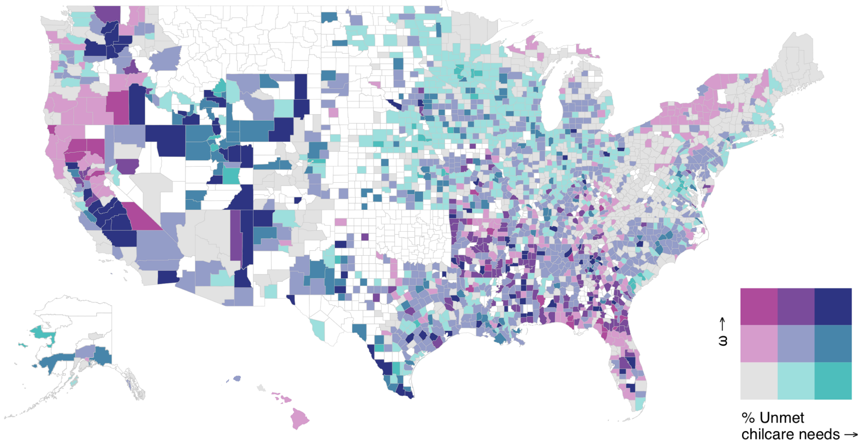

Figure S4: County-level comparison of percent unmet childcare needs and ω. Counties with confidence interval sizes in the 90th percentile or below are shown.

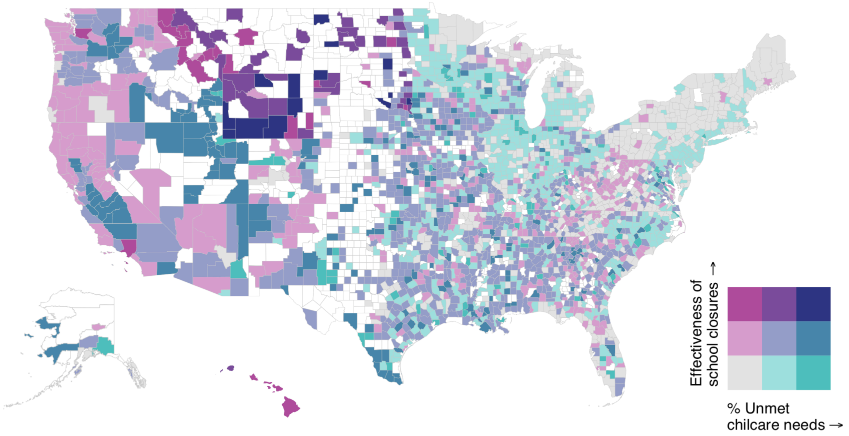

Figure S5: County-level comparison of percent of healthcare worker households with unmet child care needs and effectiveness of school closures using estimated reduction of peak ICU bed demand. Counties with confidence interval sizes in the 90th percentile or below are shown. No within-state normalization used.

Sensitivity analyses

Unmet childcare needs estimate

Population seeds	Model assumptions	All	Practitioners/Technicians	Support staff
$NHES_{0.89}$		7.5%	7.2%	7.9%
$IPUMS_{0.89}$	All	8.7%	9.1%	7.8%
	All + NE	9.2%	9.5%	8.3%
	GP	9%	9.4%	8.2%
	GP + NE	9.5%	9.9%	8.6%
	OC	12.2%	12.6%	11.3%
	OC + NE	12.5%	12.9%	11.6%
$IPUMS_{0.6}$	All	7.4%	7.6%	6.7%
	All + NE	7.8%	8%	7.1%
	GP	7.7%	7.9%	7%
	GP + NE	8.1%	8.3%	7.5%
	OC	10.4%	10.6%	9.8%
	OC + NE	10.6%	10.9%	10%

Table S2: Sensitivity analysis of unmet childcare need estimates using various population seeds. National Household Education Surveys Program (NHES) found that 50% of households had difficulty finding or could not find satisfactory child care. Integrated Public Use Microdata Series (IPUMS) are state specific seeds derived from the household structure of healthcare workers. Survey data from both the Pew Research Center and the US Census Bureau indicating that 89% of working couples rely on the mother for primary child care. To test sensitivity, we calculated unmet childcare need by assuming that 60% of working couples rely on the mother for primary child care. The NE, GP, OC, and OA model assumptions denote non-essential workers, grandparents, older children, and other adults (respectively) in the household are excluded as potential caregivers.

Transmission models

R	Hospital: SC	Hospital: SC + HH	ICU: SC	ICU: SC + HH
$State_{mean}$	10.5%	7.6%	11.7%	8.4%
$State_{lowerCI}$	53.7%	46.4%	47.7%	40.5%
$State_{upperCI}$	4.7%	3.3%	5.9%	4%
2	14.9%	10.1%	15.5%	10.8%
2.5	6.9%	4.9%	9.2%	6.4%
3	5.4%	3.8%	7.2%	4.9%
3.5	4.5%	3.1%	5.9%	4%
4	3.8%	2.7%	5%	3.4%
4.5	3.3%	2.3%	4.3%	2.9%
5	2.9%	2%	3.8%	2.6%
5.5	2.6%	1.8%	3.5%	2.3%
6	2.4%	1.6%	3.2%	2.1%

Table S3: Sensitivity analysis of transmission models under varying R_0 values and contact conditions. Estimates of the mean and 95% confidence intervals for initial reproduction number by states were retrieved on 2020-05-29. School closures (SC) reduce the risk of child-child interactions by 90%. Household (HH) interactions increase child-other age group interactions by 10%.

Model output

The glm() calls for our models and model output are below.

```
modelDiabetes <- glm(stateEstMeans~Diabetes.prevalence.raw.value+
X..65.and.older.raw.value+femalePct+pctMarried+
                X..below.18.years.of.age.raw.value+
                X..Non.Hispanic.African.American.raw.value+
                factor(state)+
                X..Hispanic.raw.value+
                X..American.Indian.and.Alaskan.Native.raw.value
                +Population.raw.value+X..Rural.raw.value,
              weights=numHCW,family=quasipoisson,
              data=regressionData)
modelCVD <- glm(stateEstMeans~cvdMortality+
X..65.and.older.raw.value+
femalePct+fmr+pctMarried+
                X..below.18.years.of.age.raw.value+
                X..Non.Hispanic.African.American.raw.value+
                factor(state)+
                X..Hispanic.raw.value+
                X..American.Indian.and.Alaskan.Native.raw.value+
                Population.raw.value+X..Rural.raw.value,
              weights=numHCW,family=quasipoisson,
              data=regressionData)
modelControls <- glm(stateEstMeans~X..Rural.raw.value+
X..65.and.older.raw.value+
femalePct+fmr+pctMarried+
                X..below.18.years.of.age.raw.value+
                X..Non.Hispanic.African.American.raw.value+
                factor(state)+
                X..Hispanic.raw.value+
                X..American.Indian.and.Alaskan.Native.raw.value+
                Population.raw.value,
              weights=numHCW,family=quasipoisson,
              data=regressionData)

summary(modelDiabetes)
summary(modelCVD)
summary(modelControls)
```

	Model 1	Model 2	Model 3	Model 4
(Intercept)	-4.25^{***}	-4.18^{***}	-4.23^{***}	-4.24^{***}
	(0.04)	(0.04)	(0.04)	(0.04)
Diabetes	0.22^{*}			
	(0.10)			
65.and.older	-0.85^{***}	-0.75^{***}	-0.82^{***}	-0.78^{***}
	(0.06)	(0.05)	(0.05)	(0.05)

	Model 1	Model 2	Model 3	Model 4
femalePct	0.67***	0.70***	0.67***	0.68***
	(0.03)	(0.03)	(0.03)	(0.03)
pctMarried	0.29***	0.22***	0.27***	0.27***
	(0.02)	(0.02)	(0.02)	(0.02)
below.18	5.57***	5.71***	5.64***	5.65***
	(0.07)	(0.07)	(0.07)	(0.07)
Non.Hispanic.African.American	0.05**	0.04*	0.05**	0.05*
	(0.02)	(0.02)	(0.02)	(0.02)
factor(state)Alaska	−0.23***	−0.26***	−0.24***	−0.24***
	(0.03)	(0.03)	(0.03)	(0.03)
factor(state)Arizona	−0.09***	−0.11***	−0.09***	−0.10***
	(0.01)	(0.01)	(0.01)	(0.01)
factor(state)Arkansas	−0.06***	−0.06***	−0.06***	−0.06***
	(0.02)	(0.02)	(0.02)	(0.02)
factor(state)California	−0.14***	−0.15***	−0.14***	−0.15***
	(0.01)	(0.01)	(0.01)	(0.01)
factor(state)Colorado	−0.01	−0.03*	−0.02	−0.02
	(0.01)	(0.01)	(0.01)	(0.01)
factor(state)Connecticut	0.03*	0.01	0.02	0.02
	(0.01)	(0.01)	(0.01)	(0.01)
factor(state)Delaware	−0.06*	−0.07**	−0.06*	−0.06**
	(0.02)	(0.02)	(0.02)	(0.02)
factor(state)DC	−0.09*	−0.10**	−0.10**	−0.10**
	(0.04)	(0.04)	(0.04)	(0.04)
factor(state)Florida	−0.03*	−0.04***	−0.03*	−0.03**
	(0.01)	(0.01)	(0.01)	(0.01)
factor(state)Georgia	−0.07***	−0.08***	−0.07***	−0.07***
	(0.01)	(0.01)	(0.01)	(0.01)
factor(state)Hawaii	−0.08***	−0.10***	−0.08***	−0.09***
	(0.02)	(0.02)	(0.02)	(0.02)
factor(state)Idaho	−0.01	−0.03	−0.02	−0.02
	(0.02)	(0.02)	(0.02)	(0.02)
factor(state)Illinois	−0.04**	−0.05***	−0.04***	−0.04***
	(0.01)	(0.01)	(0.01)	(0.01)
factor(state)Indiana	−0.04**	−0.05***	−0.04**	−0.05***
	(0.01)	(0.01)	(0.01)	(0.01)
factor(state)Iowa	0.02	−0.00	0.01	0.01
	(0.02)	(0.01)	(0.01)	(0.01)
factor(state)Kansas	−0.01	−0.03	−0.02	−0.02
	(0.01)	(0.01)	(0.01)	(0.01)
factor(state)Kentucky	−0.05***	−0.06***	−0.05***	−0.05***
	(0.01)	(0.01)	(0.01)	(0.01)
factor(state)Louisiana	0.00	−0.01	−0.00	−0.00
	(0.01)	(0.01)	(0.01)	(0.01)
factor(state)Maine	0.02	−0.01	0.01	0.01

	Model 1	Model 2	Model 3	Model 4
	(0.02)	(0.02)	(0.02)	(0.02)
factor(state)Maryland	−0.01	−0.02	−0.01	−0.01
	(0.01)	(0.01)	(0.01)	(0.01)
factor(state)Massachusetts	−0.01	−0.03*	−0.01	−0.02
	(0.01)	(0.01)	(0.01)	(0.01)
factor(state)Michigan	−0.05***	−0.06***	−0.06***	−0.06***
	(0.01)	(0.01)	(0.01)	(0.01)
factor(state)Minnesota	−0.01	−0.04**	−0.01	−0.02
	(0.01)	(0.01)	(0.01)	(0.01)
factor(state)Mississippi	−0.02	−0.02	−0.02	−0.02
	(0.02)	(0.02)	(0.02)	(0.02)
factor(state)Missouri	−0.03*	−0.04**	−0.03*	−0.03**
	(0.01)	(0.01)	(0.01)	(0.01)
factor(state)Montana	−0.02	−0.04	−0.03	−0.03
	(0.02)	(0.02)	(0.02)	(0.02)
factor(state)Nebraska	−0.00	−0.03	−0.01	−0.01
	(0.02)	(0.02)	(0.02)	(0.02)
factor(state)Nevada	−0.09***	−0.10***	−0.09***	−0.10***
	(0.02)	(0.02)	(0.02)	(0.02)
factor(state)New Hampshire	0.04*	0.02	0.04	0.04
	(0.02)	(0.02)	(0.02)	(0.02)
factor(state)New Jersey	−0.07***	−0.08***	−0.07***	−0.08***
	(0.01)	(0.01)	(0.01)	(0.01)
factor(state)New Mexico	−0.16***	−0.17***	−0.16***	−0.16***
	(0.02)	(0.02)	(0.02)	(0.02)
factor(state)New York	−0.12***	−0.13***	−0.12***	−0.13***
	(0.01)	(0.01)	(0.01)	(0.01)
factor(state)North Carolina	−0.00	−0.01	−0.00	−0.01
	(0.01)	(0.01)	(0.01)	(0.01)
factor(state)North Dakota	−0.04	−0.06*	−0.04*	−0.05*
	(0.02)	(0.02)	(0.02)	(0.02)
factor(state)Ohio	−0.06***	−0.07***	−0.06***	−0.07***
	(0.01)	(0.01)	(0.01)	(0.01)
factor(state)Oklahoma	−0.07***	−0.07***	−0.07***	−0.07***
	(0.01)	(0.01)	(0.01)	(0.01)
factor(state)Oregon	−0.04**	−0.06***	−0.05**	−0.05***
	(0.02)	(0.02)	(0.02)	(0.02)
factor(state)Pennsylvania	−0.00	−0.01	−0.00	−0.01
	(0.01)	(0.01)	(0.01)	(0.01)
factor(state)Rhode Island	−0.06**	−0.08***	−0.06**	−0.07***
	(0.02)	(0.02)	(0.02)	(0.02)
factor(state)South Carolina	0.02	0.01	0.02	0.02
	(0.01)	(0.01)	(0.01)	(0.01)
factor(state)South Dakota	0.00	−0.02	−0.00	−0.01
	(0.02)	(0.02)	(0.02)	(0.02)

	Model 1	Model 2	Model 3	Model 4
factor(state)Tennessee	-0.07^{***}	-0.07^{***}	-0.07^{***}	-0.07^{***}
	(0.01)	(0.01)	(0.01)	(0.01)
factor(state)Texas	-0.14^{***}	-0.15^{***}	-0.15^{***}	-0.15^{***}
	(0.01)	(0.01)	(0.01)	(0.01)
factor(state)Utah	-0.07^{***}	-0.09^{***}	-0.08^{***}	-0.08^{***}
	(0.02)	(0.02)	(0.02)	(0.02)
factor(state)Vermont	0.04	0.01	0.02	0.02
	(0.03)	(0.03)	(0.03)	(0.03)
factor(state)Virginia	0.01	-0.01	0.00	0.00
	(0.01)	(0.01)	(0.01)	(0.01)
factor(state)Washington	-0.07^{***}	-0.09^{***}	-0.07^{***}	-0.08^{***}
	(0.01)	(0.01)	(0.01)	(0.01)
factor(state)West Virginia	-0.08^{***}	-0.08^{***}	-0.08^{***}	-0.08^{***}
	(0.02)	(0.02)	(0.02)	(0.02)
factor(state)Wisconsin	0.03^{*}	0.01	0.02	0.02
	(0.01)	(0.01)	(0.01)	(0.01)
factor(state)Wyoming	-0.07^{*}	-0.09^{**}	-0.08^{**}	-0.08^{**}
	(0.03)	(0.03)	(0.03)	(0.03)
Hispanic	0.16^{***}	0.13^{***}	0.15^{***}	0.15^{***}
	(0.01)	(0.01)	(0.01)	(0.01)
American.Indian.Alaskan.Native	0.00	-0.00	0.01	0.00
	(0.04)	(0.04)	(0.04)	(0.04)
Population	-0.00^{***}	-0.00^{***}	-0.00^{***}	-0.00^{***}
	(0.00)	(0.00)	(0.00)	(0.00)
cvdMortality		-0.00^{***}		
		(0.00)		
Rent		0.00	0.00	0.00
		(0.00)	(0.00)	(0.00)
Rural			0.02^{*}	
			(0.01)	
AIC				
BIC				
Log Likelihood				
Deviance	1217.25	1207.57	1212.11	1216.79
Num. obs.	2857	2854	2850	2854

$^{***}p < 0.001$, $^{**}p < 0.01$, $^{*}p < 0.05$

Table S4: Regression output for models on diabetes, cardiovascular disease, percent rural, and controls.
